# "The Hospital" Medico-Sociological Supplement (No. I)

**Published:** 1911-01-14

**Authors:** 


"THE HOSPITAL"
MEDICO-SOCIOLOGICAL SUPPLEMENT.
(No. I.)

### THE PSYCHOLOGICAL DANGERS TO WOMAN IN MODERN SOCIAL DEVELOPMENTS.

By T. S. CLOUSTON, M.D., LL.D., F.R.C.P. Ed.

An Address on the "Position of Woman Actual and Ideal Society," delivered on November 28, 1910.

Looking to the social history of mankind, its movements can scarcely be said to have followed the evolutional lines of biology in living things or even the biological evolution of man. So far as is known to us, great societies, advanced in civilisation, politics, and the arts, have in the past grown up, advanced, retrograded, and disappeared. We know that in some of them profound changes in social customs, in moral sense and ethical practice, and in modes of government took place during their history, many of which seemed to lead to dangers and even to their final decay. Social developments usually have an ethical as well as a political and psychological basis. They constantly, too, have a relation to religion. Taking Egypt, for example, much as our knowledge of it has been added to of recent years, yet our picture of Egyptian social life needs much filling in; but we do know that its social conditions there changed from that of a simple family life with the women and children round the head of the house, all equally helping in domestic and other occupations—I have myself seen pictures of such a state of matters in the time of an early dynasty in a newly opened tomb—to a far more artificial social state, where the women appeared to be in a condition of subjection, like slaves. Those changes took place over and over again in the history of Egypt, marking successive periods of prosperity and of retrocession. When Queen Hatasu reigned over the land she is represented as wearing men's clothes; she initiated many movements; she ruled the land vigorously, and the country was powerful, prosperous, and advancing. When Queen Cleopatra ruled she was feminine in the worst sense, and the country was on the point of disappearing as an independent State, partly through her character and conduct. Those social changes may have been results, not causes, however. We have not the means of interpreting their full significance. The history of some of the more recent States, as, let us say, France in the seventeenth and eighteenth centuries, was accompanied by many dangers to woman in its social changes and re-adjustments.

### Society and Sex.

When social changes affect the primary qualities and functions of mankind, or even their secondary qualities and functions, such as sexual relations, marriage, the birth and care of children, the legal status of woman, the means of livelihood, the manners of the community and the modes of regarding each other by men and women, then it is clear that along with bettered and happier conditions there may also arise serious dangers. Rome is usually thought to have paid a high price for her literature and art, her riches and power, her business and world-wide commerce, in the greater separation of classes, in the greater use of slave labour, the greater dependence of the common people on the Emperor, in the relaxation of the marriage laws, and in the prevalence of what we would now call sexual offences in both sexes. The ordinary Roman citizen suffered severely in the later Empire, but the women seem to have had a worse time than the men. We do not hear of any deeds like that of Lucretia in the later times of Rome. The social feelings and changes that came about seem, in fact, to have affected the women and the family more markedly than the men.

### The Psychology of Sex.

The preliminary questions that must be referred to in treating a subject such as mine to-night are three: Is there any definite psychology of sex? And, if so, are the differences primary and fundamental, and therefore unalterable? Or are they secondary and therefore more easily affected by changes in the environment? I think it will be easy to show there are such differences of the sexes in the affective, intellectual, inhibitory, moral, and volitional faculties, and also in the instincts and the appetites, most of those being primary.

I would direct attention first to the affective faculties in my contrast and analysis, and would conjoin with those all the instincts that relate to offspring, because they are essentially emotional and affect family life, which is among the most important and practical of the relationships of the emotions. There are no definite means of testing the intensity of the emotions. As to bodily sensation, the tests by the æsthesiometer seem to show that it is somewhat less in woman than man. If the intensity of the emotions is tried by its expression in poetry, which is the literature of feeling, man has given proof of a higher depth of feeling than woman; but this may be partly because his power of literary expression has been more developed. It may be taken as fairly proved that man has more power of controlling the outward expression of emotion than woman. This, if a fact, has many social aspects. Woman, however, educated according to modern standards, is gradually acquiring more power of controlling her emotions, up to a certain point at least. This acquisition of control over the emotions and their expression is one of the psychological results that, in my judgment at least, will be a counteractive to some of the social dangers to which I shall afterwards refer. It will affect conduct, for it will give time for some consideration and thought before certain emotions are acted on. It cannot fail to have a steadying influence on woman. It will diminish unreasoning and impulsive action in the case of many women, and so give time for the *cui bono?* to come in. The emotions of anger and jealousy, and even those of satisfaction and love, will be none the worse of slower reaction than is the case in many women.

### The Instinct of Motherhood.

It is when we come to the emotions and actions connected with the maternal instinct in woman that we reach the real crux as to the effects of modern social developments on the sex. Psychologically, physiologically, and racially this is the most unique, the most wonderful, and the most important thing for mankind in the world. The continuance of life hangs on it. On it the family and home are based. From it the intensest pleasures flow. On it altruism, self-denial, self-sacrifice, and most of the unifying virtues rest. It is capable of idealising and glorifying the most common and unclean things. It can make duty a pleasure and pain a satisfaction. It can make life worth living amid poverty, disease, and even vice. In its essential essence it belongs to woman alone. Man possesses its faint analogy only. Under its imperative compulsion woman will break all the commandments in the Decalogue, and suffer little remorse for having done so. It is capable of being transformed into many other human qualities and into incredible exertion. It can make the weak strong and can stay fatigue. It can attach to itself and incorporate religious and ethical sanctions. Its power of idealisation is unlimited.

Tracing the maternal instinct up through life from its lower to its higher forms, it begins very low down indeed. Insects show it with amazing persistence, ingenuity, and what one can find no other name for than foresight. Where it strengthens and abates during, and in the intervals between, the birth and care of young it changes the whole characters of birds and animals. The timid become bold, the stupid become cunning, the silent become noisy, the night-feeder becomes a day-feeder. It is at the root of all the poetry of bird and wild animal life. Colour and song are its accompaniments. The male is infected with the reproductive enthusiasm, sharing the work and contributing the music. It is without a doubt the period of the highest physiological and psychological exaltation. Courage, pride, display, pugnacity, skill, all join in the protective and providing instincts to make it a carnival of joy. There is almost nothing it cannot do. An instinct that is so marked out by Nature must be of the very highest importance, biologically and socially. We cannot afford to neglect any facts about it. It is the most primary of all common qualities.

### The Danger of Losing It.

The question that above all others we have to devote our attention to to-night is whether there is anything in our modern social developments that does or will be likely to change, lessen, or destroy the maternal instinct. Ominous ideas are abroad on the subject. Sir James Crichton-Browne the other day boldly stated that "professional women," namely, those who had devoted themselves during their adolescence to hard study, and had attained the highest culture that modern education could give them, were having few children and did not want to have many. Statistics seem to bear this out, both in America and in this country. If this is true, there is something radically wrong with our culture or with the mode of acquiring it by our young women. Far better even have "health and ignorance" for them than this, if there is to be no choice between the two. Thirty years ago I told my audience at the Philosophical Institution that "there is no time nor place of repentance provided by Nature for some of the sins of the schoolmaster," and I asked the question, "Why should we spoil a good mother by making an ordinary grammarian?" We have greatly improved in our modes of female education since then, but have we yet arrived at the point at which the highest culture of which the woman is capable is fully consistent with the instinctive and passionate desire to have, to nurse, and to care for children, which is undoubtedly Nature's ideal of this part of woman's emotional nature, and means the continuance and salvation of a good race. Even the slightest diminution of this instinct is dangerous. Men are equally interested with women in this matter, for it is a racial one. Woman cannot fulfil her destiny if her maternal instincts are impaired. The ideals which would exalt culture above motherhood are suicidal, and should be abandoned. It will not do to say that woman should have a choice either to take up culture and intellectual work, whether it has a lessened capacity for motherhood or not, or to select domestic life. Mothers of high brain power are as much needed for an advancing race as fathers—rather more so in fact. Many people believe that the Church did harm to the race by encouraging the most sensitive and religious-minded women to shut themselves up in nunneries. The race should always be more carefully considered than the individual in any scientifically ordered community. The new science of Eugenics rests on that proposition. Nature is usually bounteous in what she provides, and we dare not needlessly throw away her gifts. We may make choices, but it must be on the lines of evolution. The fit must be selected; the unfit may be allowed to lapse in non-existence. Why should study, culture, more initiative, or more personal independence dull a woman's maternal instinct or blunt her emotional nature? I cannot see why they should necessarily do so if they are carried out on right lines and their effects carefully watched on individuals. I have often been much struck with the combined intellectual, administrative, and domestic capacities that generally prevail in the women of the Society of Friends. Perhaps they were the result of a careful selection at first. I cannot pretend to estimate scientifically the influences of a limitation of the circle of persons who by mutual consent and a certain affinity of religious instinct separated themselves to a large extent from the general community in social intercourse and marriage. The rules under which they lived seem to have made too severe a selection in mating, for in many of the Quaker families there are now signs of degeneration.

### THE "NEW WOMAN" AS MOTHER.

It is quite certain that there are many individual instances of women who had studied very hard during adolescence, acquired learning and culture, and yet were the best of mothers of large healthy families. It seems to me that it is a question of the study of the individual girl's constitution by competent observers, founding their conclusions on scientific lines which psychology, medicine, and eugenics will be able by-and-by to lay down for our guidance. Much more knowledge than we at present possess must be brought to bear on this question before we can lay down such rules as we can depend on. The problem is an extraordinarily difficult one. I think we now know enough to show that to subject a hundred girls of more than average ability to the stress of modern study and competitive examinations is attended with the risks of atrophied maternal instincts, loss of femininity, and a lessened development of "woman's ways" in a considerable number of them, who might, but for the conditions under which they passed their adolescence, have possessed these gifts. It should not be forgotten that it is during adolescence that woman first develops her greatest and most characteristic feminine as well as her moral qualities. It is according to physiological fact and law that during the development of any living organism one quality or faculty may, by undue pressure and stimulation, consume the other brain energies, which are thereby robbed of their normal power of growth. One faculty or power is fed at the expense of the others. The whole body—growth, the muscles, the general nutrition, the digestive functions, and the nerves—may thus be injured by excessive study, long hours of work, and too short sleep. The delicate graces and the subtle femininities that attract the other sex are sometimes not conserved or stimulated in the strenuous life of the student. They are, in fact, frequently neglected, and even held as despicable and unworthy. The most important of all Nature's organic cravings are thus switched off to make room for the purely mental workings of the higher brain-cells. Other ideals are substituted for the "hero" that girlhood craves for. The maternal instinct and the emotions subtilely but indissolubly connected with it are cramped and atrophied.

### THE INHIBITORY FACULTY.

The work and the position allocated to each sex have been the result of a slow evolution as society itself has become evolved. There is always a strong presumption that there must have been good reasons at some time for everything that exists, but it does not follow that changes should not be made, or even tried experimentally. The great facts of former evolutions in living beings have not come about as the result of man's reason or planning. But the more recent evolutionary changes in society and in ideas seem, many of them, to be contrary to the lines of Nature's evolutionary processes. The weak are not allowed to die, and the unfit at the present time, it is believed, may become the fit through right environment. Gentleness, sympathy, help to the helpless, condemnation of neglect and cruelty, the removal of ignorance and its results, severe limitations of power, the responsibility of every man and woman for the condition of their fellows, theories of the political and social equality of all men and women, and of the innate equality of the sexes, are now regarded as strictly evolutionary. A new human evolution has, in fact, arisen on a humanitarian, ethical, and religious basis. Are the various movements whose aim is now to alter the social, economic, and legal positions hitherto held by women on the lines of this new evolution? Are there any risks in this to woman's innate nature, and, if so, what are they? Further, can the gains be obtained without losses? Above all, can the risks be obviated by any modification of the present movements, founding our conclusions on results of the experiments that have been made up to this time? I firmly believe the most serious risks can be avoided by candidly admitting the evil results that have already showed themselves, and by studying woman's essential attributes so that the new evolution may be run on safe lines. It is primarily a scientific question, and must be solved by the methods of modern science. I need hardly say I do not restrict "science" to mere "natural phenomena," but include in it sociology, psychology, and ethics. I would like to include under science politics and religion, but the time for that has not yet come.

The inhibitory faculty in woman is weaker than in man, but it may be gradually increased by the right kind of education and experience. It will take many generations to do so, however. Being the highest of the mental powers, and the one on which the moral sense depends in its effects on conduct, it will be slow and gradual in its gains. Self-control is the last result of evolution, the last to come to perfection in the adolescent, the last and best result of civilisation. It is also the first to go in the dissolution of mind in old age and in mental disease, and in societies that are on the down grade. A certain lack of it is, I fear, almost expected in woman, and the highest degrees of it are not commonly looked for in her. Where it is seen we instinctively call a woman "masculine," as in the few who are not prone to yield in conduct to emotion, instinct, and impulse.

### THE EMOTIONAL SIDE.

In sympathy, in fancy, and even in some departments of the higher imagination and in the dramatic art, woman clearly exceeds man and needs no cautions as to the possible effects of modern social developments. It is man who needs more evolution in those qualities.

When we compare the judging, the reasoning faculty in man and woman, we are met with the usual and to a considerable extent true and general belief that man comes to his conclusions by searching for and observing facts, woman to a large extent by "instinct." Bacon, the great originator of the scientific method of reasoning by induction, was the furthest from the feminine type of any man of his time. No woman has ever attained that type of mind, but Bacon's mother is described as a woman of powerful and strong religious feelings. When woman by education and evolution has been able to learn and to practise the Baconian method of reasoning—if that is possible—will she lose her present faculty of coming to conclusions by instinct? I believe she will do so to a large extent. I am not at all sure that the gain would quite fully make up for the loss. Looking at human conduct, it is regulated quite as much by instinct as by reason. Mistakes are made by both processes, but not much more frequently by one than the other.

### Social Instincts.

The social instincts are sharper in woman than man. There are no records of women hermits and misers. I think the higher education tends in some women to lessen the craving and necessity for social intercourse. Books, reflection, and congenial work with them take the place of the society of their fellow creatures. They become somewhat intolerant of fools and bores with their "aimless chatter"—a doubtful gain in ordinary life as we find it. Women of education should try and conserve to some extent this faculty of being able to chat about nothing in particular and to enjoy it, if their own happiness and that of others is to be considered. Otherwise they will not be good with children or enjoy their society.

There are two things that have played a great part in the evolution of civilisation—chivalry in the man and modesty in the woman. Chivalry is a secondary quality; modesty is a primary quality. The first is of great psychological, ethical, and evolutional interest. The experience of most people who have mingled in modern society is, I believe, that now woman is frequently not talked of and often not treated by men with that form of practical idealism which is the basis of chivalry. On the other hand, some matters are now openly discussed by women in much-read books and in personal intercourse which would have shocked modest women fifty years ago. Both those tendencies are absolutely evil, I most strongly believe. They are in the direction of reversion, not of evolution, sociologically and morally. They should be strenuously combated and avoided by all who desire the upward movement of both sexes.

### General Conclusions.

Women have hitherto been far stronger in the great and educative faculty of imitation than the other sex. It is one of their strongest points psychologically. Many of the professional and highly educated women that I have met talk of it somewhat scornfully as a lower mental characteristic that should be repressed. The chief imitativeness that I think needs guarding against by woman is that of the student trying to walk, dress, speak, and smoke like a young man.

The most important general conclusions for discussion I would put down in regard to this question are the following:—

1. The special qualities of man and woman are complementary, both being needed for the evolution of the ideal society of the future.

2. Changes should be gone about in a scientific spirit, experimentally, guardedly, and taking into account the primary characteristics of woman as distinguished from her secondary qualities.

3. Equality of the sexes should be limited to opportunity, legal status, and social position, and should not be attempted as to faculty and instinct.

4. The lines on which civilised woman has evolved in the past should be carefully taken into account in any new schemes of the future.

5. No general schemes or systems can be universally applied with safety in regard to woman's education. Special study of each individual and of the effects of any system or change of system on the individual woman by competent observers is needed if the best results are to be secured.

### THE FASCINATION OF DANGER.

Among the summaries of the events of the year which always fill our newspapers at the end of December, special articles were devoted to the progress of the latest of sports—aviation. The tale was told of feats which only a year ago would have been deemed impossible. But towards the end of the tale came the statement: "The deaths during the year total up to a distressing number. Of aviators who succumbed to injuries received in accidents to their machines there have been twenty-nine." Which means that in a sport that, from the risks involved and also from the inevitable expense of taking it up, is followed by comparatively few, aviators have been killed at the rate of rather more than one each fortnight.

Yet there is no prospect of aviation being abandoned by its votaries. Daniel Kinet was killed in Belgium, but even his own brother went on flying after the accident. Mr. C. S. Rolls was killed on the second day of the Bournemouth Meeting, but the meeting was not abandoned, and Messrs. Rawlinson and Alan Boyle were injured subsequently. Accidents have increased, but aviators go on until they meet with an accident, and tyros are still learning to fly and trying to win their pilot certificates. What can account for this, in an age in which ease has become a fine art, except the survival of a primitive instinct which loves danger for its own sake, and which seeks to make possible that which all the world has decreed to be impossible? To do something which no one else has done—to run a risk which has been fatal to others—can it be denied that in this thought there is a fascination for those who are among the best of mankind?

There is vanity in it, doubtless; but it is not all vanity. There is folly in it; but it is not all foolishness. There is ambition in it; but it is not all ambition. It is an unreasoning instinct—for in most of the cases reason would act as a danger-signal—which compels a man to do something more than has ever been done before. It may mean victory or it may mean death; but the man who considers too closely the possibility of death never achieves the victory. Though perhaps it often flies off at a tangent, and wastes its energy on achievements which are valueless, it is the spirit of progress, the spirit which has won for us all our civilisation; and even if that civilisation tends to make the danger risks of primitive life unnecessary, it is a spirit whch will survive to make new risks for itself, to achieve new records, and thereby to make possible a yet higher degree of civilisation than has been yet attained.

### PARENTAL ALCOHOLISM AND HEREDITY.

### A MEDICO-SOCIOLOGICAL DISCUSSION.

At the ordinary meeting of the Society for the Study of Inebriety, held at the rooms of the Medical Society of London on Tuesday, January 10, the subject for discussion was "The Influence of Parental Alcoholism on the Physique and Ability of Offspring." It was opened by the President of the Society, Dr. Theo. B. Hyslop, F.R.C.P.

### The Opening Paper.

Dr. Hyslop, in his opening, reminded his hearers that the subject had been treated from many and diverse points of view, but the aim of the Society was to arrive at the whole truth. Admittedly discussions on alcohol were sometimes characterised by extravagances, but personal conflicts did no harm so long as they did not obscure the matter discussed. It behoved students of the question to divest themselves for the time being of all their previous notions and beliefs, and endeavour to hark back to the beginning, so as to build upon what was really sound. The Society was in sympathy with the aims of the Eugenics Laboratory, but did not approve of its methods; and he hoped that friendly collaboration would enable students to avail themselves of the vast amount of earnest work which had been done by the best brains of the day. The problem was not provable nor capable of advancement by rhetoric; indeed it might not be solvable even by statistics. The problem could be stated as follows: "Does parental alcoholism—apart from parental degeneracy, which, together with a tendency to alcoholism, is heritable—influence the physique and ability of offspring?" Side issues must be eliminated, and one should equalise or exclude such variations in environment as might possibly affect the germplasm of the parents or the offspring independent of the influence of alcohol itself. But it was practically impossible to find a definite uniformity of phenomena on which to base statistics which should be accurate in every particular. All variations in environment could not be excluded, nor could the possibility of the intensification of degeneracy in successive generations due to the continuance of the same or different environmental defects. In order to better define standards of parental degeneracy and alcoholism, one must note the sex, age at incidence, and evidences of inheritance, as well as the nature and degree of the defect.

### The Work of the Galton Laboratory.

The Galton Laboratory had brought into prominence the association of alcoholism and degeneration, as contrasted with the causation. But the question as to which came first had not yet received the necessary attention, and as an asylum physician he was confronted with the difficulty of deciding which was cause and which effect. Thus it would be seen how difficult it was to find reliable data on which to base statistics. The tendency to alcoholism may be latent in the parent until accidentally revealed, after transmission of the tendency to the child. At first he believed that parental alcoholism should invariably precede the birth of the child to transmit the taint, but he now saw that such a stipulation would lead to the suppression of half the truth, and provide a source of error unless one distinguished between parental alcoholism *per se* and parental degeneration *plus* a psycho-neurotic tendency to alcoholism. The assertion that alcohol at festivals was the source of imbecility in the offspring had not, he contended, been proved, though probably more conceptions took place at such times.

### Intra-Uterine Changes.

Moreover, little was yet known of the influences, alcoholic or otherwise, which determined conception. With regard to the point concerning the influence on the child when alcoholism was present in the mother, as compared with its presence in the father, the matter was not of great importance, compared with the researches in which small quantities of alcohol were given to animals, for probably the offspring had not inherited a parental neurosis or psychosis, to which the effects of alcohol had been added and transmitted to the offspring. He accepted Laitinen's statement that the number of young of those animals which received alcohol was somewhat larger, but they were much weaker than in the case of the young of animals not treated with alcohol. For a long time he, Dr. Hyslop, had attributed mental and physical defects to parental alcoholism, but careful study of the investigations of the members of the Eugenics Laboratory led him to try to discard his previous conceptions; and he could not convince himself that in any one case other factors than alcohol had also been at work; there might have been a direct inheritance of a neuro-psychosis as well as alcohol. Instances of germ-plasm in the stock were instances of heredity pure and simple, and they ought, for the solution of the problem, to be rigidly excluded. Yet that might result in the elucidation of but half the truth. His experience at present led him to the belief that parental alcoholism accentuated the downward trend, and that with each successive generation the period of exemption from alcoholism and degeneracy was shortened, so that the offspring became alcoholic or degenerate at a relatively earlier age.

### Alcohol as a Factor in Degeneracy.

In families prone to degeneracy alcohol appeared to put the finishing touches; it would readily set alight and determine the existence of a neurosis or psychosis which might otherwise be on the wane; and it also seemed to render the offspring more liable to suffer from the transmission of such degeneracy. The last report from the Galton Laboratory claimed that alcohol was, in its pernicious forms, consequent on, and not antecedent to, mental defect, and that might be true. "One man's food another man's poison" referred to alcohol, and it was difficult to differentiate the degrees of alcoholism. In asylum practice it was comparatively seldom that one found the various lessons so common to alcoholism in the sane. Possibly that was because alcohol selected the least stable of the bodily systems in a given individual. The system might be, by its inherent weakness, more susceptible to attack, or it might be less able to eliminate from its substance the effects of toxic action. It would aid the solution of the problem if there could be found well-defined and accurate instances in which the defect in the child could be proved to be due to parental alcoholism without the aid of any other causal factor. Until that could be done, and until such instances could be multiplied, any statistics could only assist in the making of hazardous half-guesses, which might lead to misinterpretation and the spreading of doctrines and lines of action which might be for the good, but also possibly for the ill, of the community.

### A Guarded Opinion.

Mrs. Scharlieb, in response to the President's invitation, said she would not like to advance any views on the subject after the point of view so ably presented by Dr. Hyslop. She would prefer to think the matter over, but her view had always been that alcoholism in the parent was likely to lead to degeneracy and feeble-mindedness and crime in the offspring.

### Sir Victor Horsley's Conclusions.

Sir Victor Horsley, F.R.S., suggested that someone from the Galton Laboratory should speak first, but he was informed that none of those invited from that institution had come. He regretted that the discussion was likely, on that account, to be one-sided. The position of the subject which Dr. Hyslop had placed before the meeting was totally different from that which the Galton Laboratory advanced; the former was a philosophic consideration of the subject, and it showed that the matter could be considered from two points of view, viz. as to whether children did suffer directly from alcoholism in the parent, or whether they might be simply exhibiting the inheritance of some psycho-neurosis. He (Sir Victor) proposed to deal only with the question of parental alcoholism *per se*, a point on which much scientific work had already been done. And that work all went to show, until last May, that alcoholism in the parent was followed by adverse consequences to the offspring.

### The Pearson-Elderton Monograph.

But in May last the world was much startled by the paper published by Miss Elderton and Prof. Karl Pearson—startled from the sociological and toxicological points of view; sociologically because the memoir summed itself up in two statements that, sociologically speaking, drinking working men were as good as others, physically and mentally as good, if not a little better. The toxicological statement was that examination of children of school age showed that those children were not suffering from any poison which the parents had taken. What he (Sir Victor) proposed to lay before the Society by way of fact Dr. Mary Sturge was responsible for as well as himself. Yet in all their letters to the *British Medical Journal* the Galton Laboratory left out all mention of his colleague, Dr. Mary Sturge, though her work on the subject had been proceeding for the last twenty years. When the Memoir No. X. was first published, Prof. Marshall at once pointed out in the *Times* that the treatment of the data by Prof. Pearson as to whether the drunken workman earned as good wages as the sober workman was not correct, statistically speaking. In the next issue of the *British Medical Journal* Dr. Mary Sturge and he would show that Miss Elderton and Prof. Pearson had treated the Edinburgh statistics in a most unjustifiable manner.

### An Attack on Professor Pearson's Data.

Prof. Pearson published in the *Times* what he called a wage table; he grouped the trades of the parents of the children together, and then attached to each trade a definite wage. Then he gave a statement of the number of the parents who were sober and the number who drank alcohol. Those statistics were absolutely untrustworthy, as proved by Mr. Scanes, in the *Statistical Journal*, last summer and last December. It would be found that Prof. Pearson had put down, say, ten carpenters, and put to them an average wage. It was scarcely credible, but what the professor derived his information from was not the schedules of the Edinburgh report, but the table which the Edinburgh workers calculated by repeated investigation; not for ten cases, because the data were insufficient, but for six cases. Prof. Pearson actually took that figure and gave it to all the ten. But, as would be seen in the paper on Saturday, the professor added to the table men who did not exist. A case in point was that of railway porters. Mr. Scanes pointed out that Prof. Pearson said there were nine railway porters—two were sober and seven drank. But as not more than two railway porters were mentioned anywhere in the report, Prof. Pearson invented seven and gave them the accurate wage rate; not that mentioned in the schedules of the report, but one in which the Edinburgh table was misprinted ten shillings higher than it really was. Prof. Pearson stated all those railway porters to be earning 28s. a week, whereas the fact was there was one railway porter and he was earning 18s. a week.

### Statistics.

The wholesale manipulation of the Edinburgh statistics was a discredit to statistical science. When the persons who drank were put on the opposite side to those who were relatively sober, it was found that the sober workman earned from 15 per cent. to 20 per cent. more than did the drunken worker. Thus he did not hesitate to characterise the generalisation in Prof. Karl Pearson's paper as absolutely untrue. With regard to the point as to parental alcoholism *per se* and its influence on the children, the curves and mathematical tables in that memoir were absolute deception—it was the only word he could apply to them—because they professed to be accurate mathematical determinations of the conditions of the children according to whether the parents were teetotallers, sober people, or excessive drinkers in various degrees. As Prof. Marshall pointed out last July, it was essential to know whether the parents were alcoholic or not; in other words, until the incidence of parental alcohol was determined, no one knew to what category those children belonged. Yet Prof. Pearson had assumed that he knew; the facts were not in the Edinburgh Report, and no one knew anything about Miss Dendy's figures—they ought to have been published long ago. Naturally, it was absolutely necessary to know whether a parent was alcoholic or not. Dr. Sturge and he had for several weeks written controversial letters in the *British Medical Journal* to extract from Prof. Karl Pearson essential facts, but each week he put them off with some statement about the number of children the alcoholic might have. This week Dr. Sturge and he had again asked for figures, and if he did not publish the figures in the *Journal*, they invited the readers not to believe him at all.

### Teetotalism.

He (Sir Victor) mentioned the word "teetotal," and he did so for a definite reason. In their Memoir, Miss Elderton and Prof Pearson said that the number of teetotalers was so small—which was true, as only nineteen teetotalers could be found in 781 families—that they had merged the teetotalers into the figures for sober people, and they could not deal with them separately. But they had dealt with them separately, and not only in their tables at the end of their paper, but even in the current issue of the *British Medical Journal* there was a new table published by Prof. Pearson to depreciate teetotalers, in which he compared the children of teetotalers with the children of alcoholics of various degrees, and put at the top of the series "1,000 children." The facts were that perhaps he had twenty-five children to put into the teetotal column. In his Memoir he said the number was too small to deal with, but later he dealt with them. That was not scientific good faith. The whole of that Memoir was, as Sir Thomas Whittaker said, a mathematical superstructure on a rotten foundation, and the rottenness was demonstrated increasingly the more it was looked into.

### Insanity and Alcoholism.

Had there been time he would have liked to deal with the point that the asylum evidence seemed to support the Galton theorists; but their last Memoir on extreme alcoholism had nothing to do with it. But the evidence of Dr. Mott, who did know what he was talking about, must be gone over from the point of view of the person who was admitted into an asylum and remained there a number of years, and who would not have the same acute visceral lesions as would the person who died from acute alcoholism. In national degeneration there were many factors, and the Society for the Study of Inebrity had the merit of investigating one of them—alcohol. And its work would continue, and would not be hampered by the publication of memoirs of the kind he had referred to, which were naturally welcomed by certain interested persons in the community, and, he was glad to say, they would not go without an answer.

### Some General Experiences.

Mr. M. Ryley related a number of instances in his own experience which showed that alcoholism was a transmitted taint. In one case the whole of the female branch of a family were intemperate, following the vice in their father, and they all killed themselves. In a similar clear case the lady was almost a degenerate, and was bereft of memory. When the two elder members of a family were conceived the father was very fond of stimulant, and they were addicted to alcohol, but the younger members of the family were abstainers. Another case was that of a judge who took much alcohol for some years before he died, and his daughter was constantly under treatment in various ways for alcoholism, and eventually died from it. It had been urged by people that alcoholism was not hereditary because it did not show itself until drinking commenced. That was about the same as saying that gunpowder was not dangerous because it was inert until a lighted match was applied.

### Dr. Sullivan's Conclusions.

Dr. Sullivan (a prison physician) said the initial difficulty was to understand what was meant by alcoholism when speaking of it as a cause of degeneracy in the offspring. Chronic alcoholic poisoning caused more or less durable disorders, and, in extreme cases, led to death. To be effective in the next generation it must have existed in the parent some time before conception. Chronicity was the keynote, and the most illuminating material was that in which ordinary people had had normal healthy children, had later taken to excessive indulgence in alcohol, and then had defective children. Prof. Pearson's idea of alcoholism, that it was when the person drank more than was good for him economically or for the proper upkeep of the home, would not do. Prof. Pearson was very apt at criticising other people's evidence, yet on such a basis he said that in the Edinburgh school 34 per cent. of the mothers of the boys and 64 per cent. of the fathers were suffering from chronic alcoholism. There was a residue of medical evidence showing filial defect following alcoholism in the parent, but there was great need for experimental corroboration. Prof. Pearson assumed that drunkenness was evidence of chronic alcoholism—quite a fallacy. A much surer basis was the occurrence of delirium tremens, and it had been shown by statistics in 1906 that 60 per cent. of the males and 30 per cent. of the females who had delirium tremens had never been convicted for drunkenness or anything else. Prof. Pearson suffered from the disabilities of people who dealt with statements of which they had not first-hand knowledge. It was in the children of those who drank industrially rather than convivially that evidence of defect due to alcohol would be found.

### Experience versus Theory.

Dr. Claude Taylor contended that experience was worth something, for it observed life over considerable periods and with many ups and downs. Prof. Pearson's conclusions might lead to unjustifiable measures in the matter of segregation, many of the persons apparently not being far removed from the normal. He was not prepared to base conclusions on statistics which had been shown to be in so many ways unwarranted.

The Rev. Canon Horsley thought that long association with people of a locality counted for more than a study of statistics. The children of tipplers were invariably undersized, and the earlier children of a marriage were stronger than those born after drinking habits had become more confirmed. Moreover, the children of alcoholic parents showed increased excitability and loss of self-control at puberty.

### Dr. Hyslop's Reply.

Dr. Hyslop, in his reply, said that most medical men did not know what the problem was. Gratitude was due to Sir Victor Horsley and Dr. Mary Sturge for preventing statistics founded on wrong data and imperfectly digested being palmed off on the public without contradiction. He agreed that experience was worth more than statistics, for the latter paved the road to delusion. The great lesson which had been learned—and he emphatically repeated it—was that no person who had the slightest predisposition to any psychoneurosis should touch alcohol. He appreciated Dr. Laitinen's work very much, and he did not quarrel with his statistics, but he considered the time was not yet ripe for the acceptance of all his data. Moreover, Laitinen did not differentiate between the purely alcoholic parent and the alcoholic parent who also had a degenerative tendency.

### CRIMINALS' BRAINS.

Much has been written about the external aspects of the criminal; so much, indeed, that ordinary people are almost afraid to look at their nearest and dearest lest the shape of the cranium, the position of eye or ear, the curve of the nose, may indicate some criminal tendency. Doubtless we are all, under certain circumstances, potential criminals. There are moments when we wish the death of any one who has, or whom we imagine to have, wronged us. If envying our neighbours' superior prosperity be a potential theft, as strict moralists tell us, then none of us are quite guiltless of robbery. But that is a different matter from carrying our vague envies and enmities into actual murder and theft. The man who does that is clearly one in whom the balancing characteristics of justice and pity on the higher plane, and caution and cowardice on the lower, are evidently lacking. It would be interesting to know if the truly criminal characteristics —those which find vent in action—are indeed to be read by the formation and convolutions of the brain. The inquiry would certainly have a scientific value, and it might have a practical one also. If it were proved that the brains of criminals differed in a marked degree from those of the normal subjects which are found in our dissecting-rooms, it would be a strong argument in favour of those who hold that crime is only a form of lunacy, and should not meet with severe punishment, but rather with such merciful restraint as we give to lunatics. On the other hand, if it were shown that the criminal is a man of normal capacity, who has yielded of his own will to those impulses of hate and jealousy to which we are all subject, it would form a strong argument for the severer punishment of crime. Philosophical generalisation is the rule with both humanitarians and penalists, and the results of their philosophy are not altogether successful. A little pathological anatomy might help them to find a common basis of agreement, and redeem our penal code from the reproach of being at once vindictive and futile.

